# Experimental characterisation of the bound acoustic surface modes supported by honeycomb and hexagonal hole arrays

**DOI:** 10.1038/s41598-019-50446-z

**Published:** 2019-10-31

**Authors:** Timothy A. Starkey, Vicky Kyrimi, Gareth P. Ward, J. Roy Sambles, Alastair P. Hibbins

**Affiliations:** University of Exeter, Electromagnetic and Acoustic Materials Group, Department of Physics and Astronomy, Exeter, EX4 4QL United Kingdom

**Keywords:** Surfaces, interfaces and thin films, Acoustics

## Abstract

The Dirac point and associated linear dispersion exhibited in the band structure of bound (non-radiative) acoustic surface modes supported on a honeycomb array of holes is explored. An aluminium plate with a honeycomb lattice of periodic sub-wavelength perforations is characterised by local pressure field measurements above the sample surface to obtain the full band-structure of bound modes. The local pressure fields of the bound modes at the *K* and *M* symmetry points are imaged, and the losses at frequencies near the Dirac frequency are shown to increase monotonically as the mode travels through the *K* point at the Dirac frequency on the honeycomb lattice. Results are contrasted with those from a simple hexagonal array of similar holes, and both experimentally obtained dispersion relations are shown to agree well with the predictions of a numerical model.

## Introduction

The unusual dispersion of the electronic bands in graphene has inspired a wealth of studies examining the analogous behaviour of classical waves propagating on surfaces or through materials comprising structures with similar symmetries, so-called ‘artificial graphene’ structures. Although the behaviour of electrons, optical waves, and elastic waves is described by scalar, vector, and tensor waves respectively, studies exploring electronic^1^, photonic^2–4^, polaritonic^5^, phononic^6–8^, and plasmonic systems^9–11^, all exhibit linearity in their dispersion near high symmetry points in momentum space. An example of a 2D phononic crystal that exhibits regions of linear dispersion is a triangular lattice of iron rods embedded in a water host^12^. In that study, the gradients of the linear dispersion at multiple high symmetry points (at Γ and *K*) were found to be proportional to the strength of coupling between degenerate Bloch states. Recently, researchers have reported the existence of both acoustic Dirac (deterministic) and Dirac-like points at the Brillouin zone centre^13–15^, and this phenomenon has been highlighted as important for the realisation of either zero phase change propagation^13^ or topologically protected edge states for acoustic waves^12,15,16^.

In recent years, following the discovery of ‘spoof’ surface plasmons (SSPs) in the electromagnetic domain^17,18^, bound acoustic surface waves with behaviour analogous to that of SSPs have received considerable attention^19^. Bound acoustic surface waves on sculpted surfaces arise from the interference between localised (evanescent) and propagating fields, and have been used to demonstrate applications such as super-resolution^20^ and deep-subwavelength focusing^21^. On these structures, the pressure field profile decays exponentially in the direction normal to the surface, and the magnitude of momentum supported in the direction parallel to the surface significantly exceeds the momentum of sound in air.

Some structures supporting bound surface states display an interesting feature in their dispersion relation; a linear crossing, or Dirac point, lying outside the sound line (defined by the dispersion of a free space sound wave). The existence of acoustic Dirac points at the Brillouin zone edges was first studied theoretically by Zhong *et al*.^22^, and modes that propagate along the zig-zag edge were numerically demonstrated. Experimentally observed Dirac cone acoustic surface waves at the Brillouin zone corners were first reported by Torrent *et al*.^23^, who studied the dispersion relation of an acoustically rigid surface with cylindrical cavities drilled in a honeycomb lattice using a phase-delay measurement. The presence of Dirac points in the dispersion relation of the acoustic surface waves supported by this surface was demonstrated, with comparisons made between experiment and theory.

**Figure 1:**
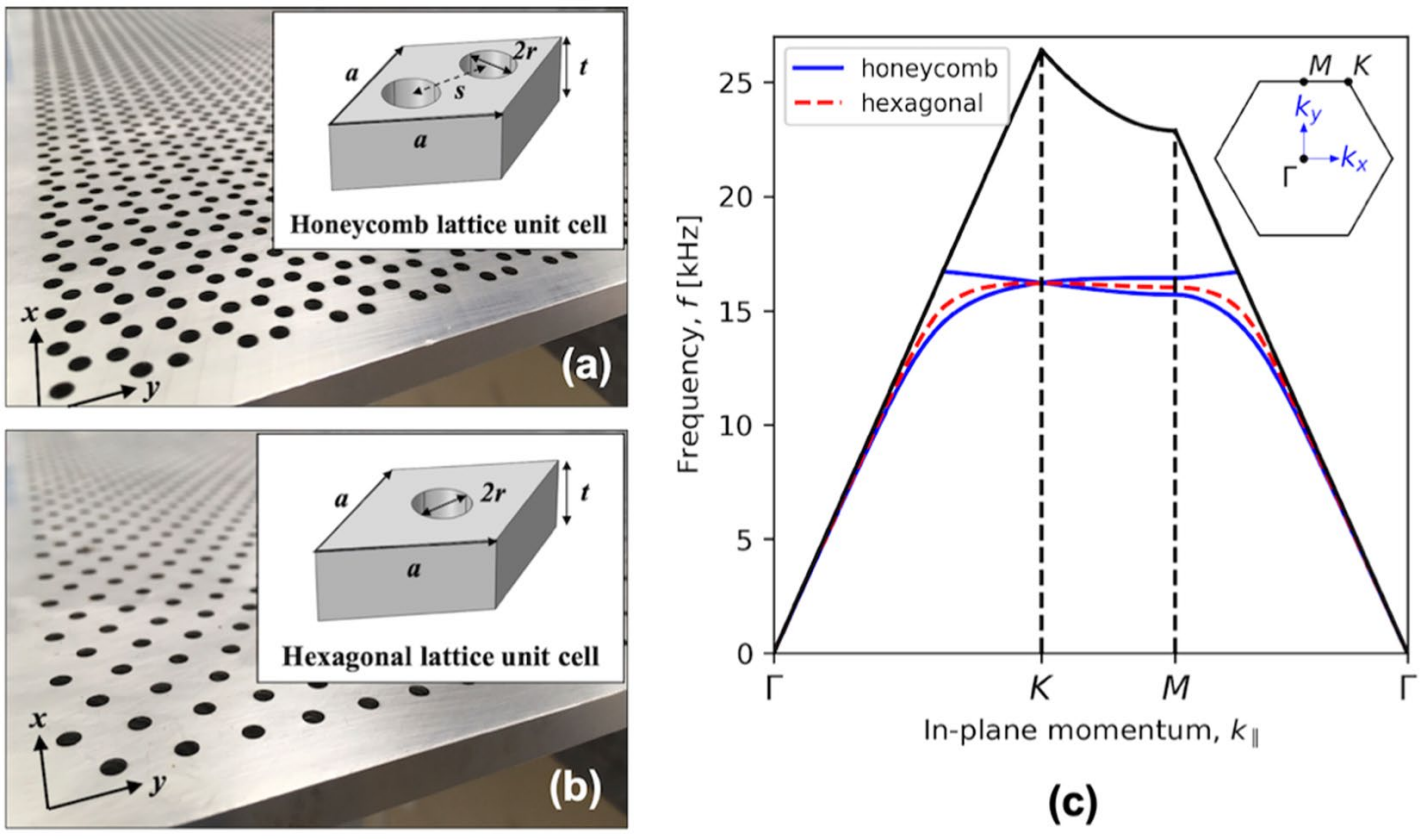
(**a,b**) Photographs of the aluminium samples and unit cell schematics (inset) for (**a)** the honeycomb array, and (**b**) the hexagonal array explored in the numerical modelling and experiments. The nearest neighbour distance between holes for the honeycomb sample and the hexagonal sample is *s* = 5 mm and 8.66 mm (not shown), respectively. The hole radius, *r* = 1.5 mm, and the mean plate thickness (length of the open cavity), *t* = 8.0 ± 0.1 mm, are the same for both samples. (**c**) Numerically (FEM) calculated band structure of the acoustic surface waves for samples in (**a**,**b**). The sound line, calculated for sound propagating with velocity, *c* = 342 ms^−1^, is shown as a solid black line, and the vertical dashed lines indicate the 1^st^ Brillouin zone edge at the *K* and *M* points. Inset: shows the points of high symmetry in momentum space for both lattices.

In this work, using experimental techniques and numerical modelling, we explore, in the vicinity of the deterministic Dirac points, the dispersion of acoustic surface waves supported by a solid plate patterned with a honeycomb array of ‘through’ holes (Fig. 1), and compare with results from a similar hexagonal array. By measuring the acoustic pressure field across these structured surfaces, we obtain a full picture of the dispersion of the supported surface waves.

## Results

Here, we study the lowest order surface modes, for which there are two for the honeycomb and one for the hexagonal array, as determined by the available number of degrees of freedom of the unit cell (i.e. here this corresponds to the holes per unit cell). These surface modes are associated with the diffraction between cavity resonances supported by quantisation of the pressure field in the cavity depth; here, the cavity resonances have the approximate character of a half-wavelength pipe modes (*λ*/2 resonance) within the open holes, and are coupled by diffraction near their resonant frequency producing an eigenmode that is strongly localised to the top and bottom interface of the structured surface, which decays exponentially from the surface. This mode is termed an Acoustic Surface Wave (ASW) since it is both trapped, because it has too much momentum to radiate into free space, and is wholly mediated by the air above the sample (unlike a Surface Acoustic Wave (SAW))^24–26^.

Samples were designed to have acoustic modes that exist within the audible frequency range, and for surface mode wavelengths that make measurements feasible on a desktop scale. The samples studied, shown in Fig. 1, have holes milled through a plate with cylinder radius, *r* = 1.5 mm, hexagonal lattice periodicity, *a* = 8.66 mm, and plate thickness *t* = 8.0 mm. The dispersion relations as a function of in-plane momentum (${k}_{||}=\sqrt{{k}_{x}^{2}+{k}_{y}^{2}}$, where *k*_*x*|*y*_ is the momentum in *x*|*y* direction), obtained using Finite Element Method (FEM) eigenvalue numerical simulations (see *Methods*), between points of high symmetry (Γ, *K*, and *M*) are shown in Fig. 1(c). These eigenvalue solutions show both honeycomb and hexagonal hole array structures support bound acoustic surface waves, as indicated by solutions that are bound within the sound line (black line), i.e. in the non-radiative regime. In the vicinity of each *K* symmetry point, the dispersion curve for the honeycomb structure is linear and exhibits a Dirac crossing of two surface modes, whilst at the *M* point a band gap exists. For the hexagonal array, the dispersion shows a different behaviour; only one surface mode crosses both the *K* and *M* symmetry points at frequencies close to the crossing point of the honeycomb array. Both arrays show relatively flat dispersion in the proximity of *K* point for the structure dimensions considered here.

To experimentally obtain the full dispersion, an acoustic pressure field mapping technique was used; a loudspeaker source fixed at the centre of the sample generated a pulse which was measured by a detector on the other side of the sample mounted on a translation stage (for further details see *Methods* and Fig. S1). The point-like acoustic excitation, which is diffracted through the hole at the centre of the scan area, excites the surface modes due to the overlap of spectral- and wavevector- spectra of source and surface mode. By scanning the detector over the sample surface the data collected forms a 2D map of the signal as a function of time, from which temporal and spatial Fourier analysis produce full dispersion information. Figure S2 displays an example of the time-space map of an acoustic pulse propagating across the sample surface and examples of the pressure field distribution obtained after the temporal signals are Fourier transformed.

**Figure 2:**
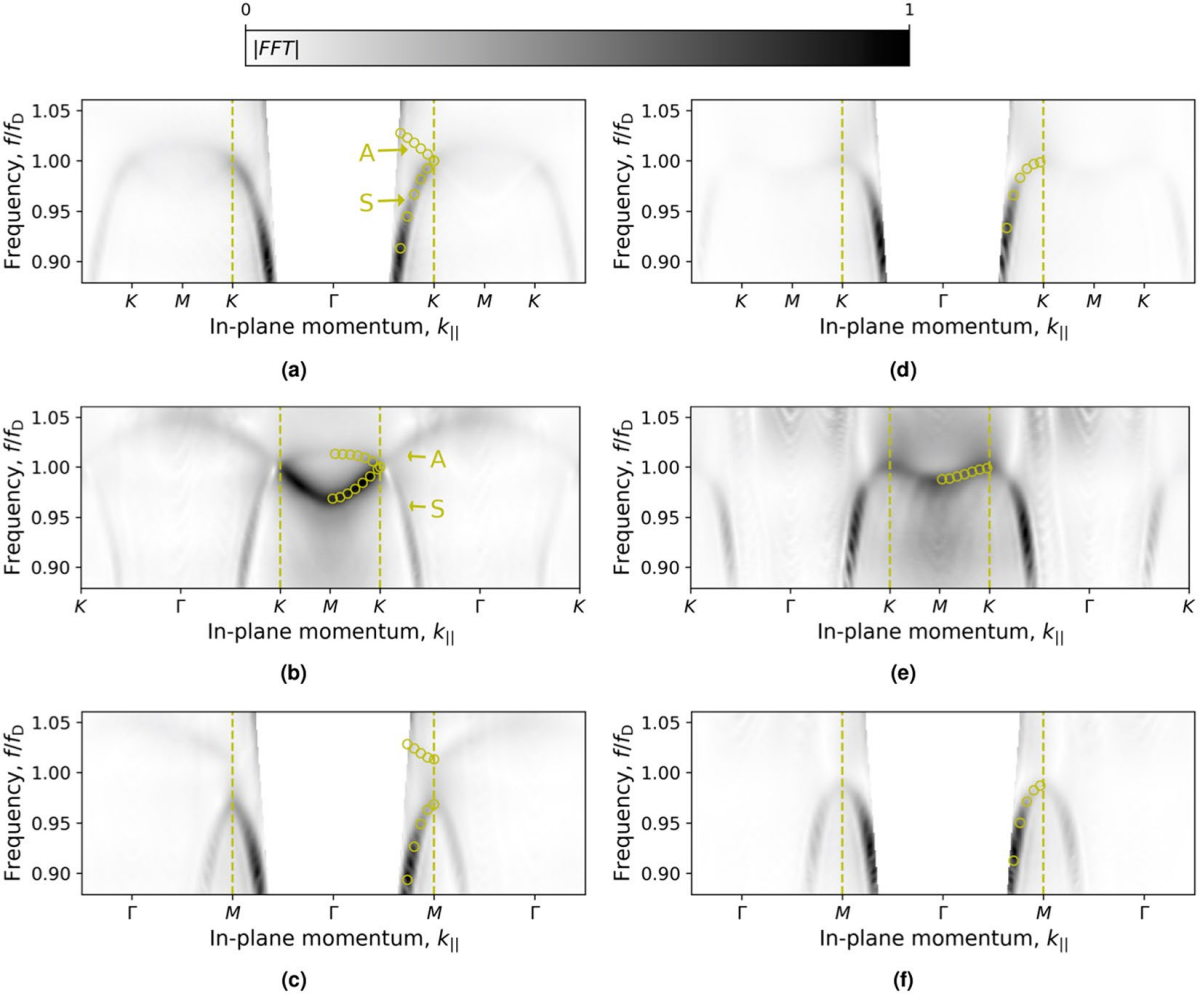
Dispersion measurements and numerical predictions for the honeycomb, left panels (a–c), and hexagonal structure, right panels (d–f). Measured data (greyscale) is shown as the Fourier intensity, and the simulated data (points) is shown for positive momenta in the first Brillouin zone (indicated by the dashed lines). Top panels show dispersion on Γ-*K* plane (with *k*_y_ = 0 fixed, and *k*_x_ varying); middle panels show dispersion on *M*-*K* plane (for ${k}_{{\rm{y}}}=-\,2\pi /a\sqrt{3}$, and *k*_x_ varying); bottom panels show dispersion on the Γ-*M* plane (with *k*_x_ = 0 fixed, and *k*_y_ varying). White areas mask the radiative regime (i.e. from Γ to the sound line), and the labels *A* (anti-symmetric) and *S* (symmetric) on panels (a,b) denote the mode pressure field symmetry with respect to the mode propagation direction.

Figure 2 displays the measured dispersion curves in the Γ-*K*, *M*-*K*, and Γ-*M* directions in momentum space for both the honeycomb array and the hexagonal array. Experimental data are compared to numerical simulations for the range of momenta lying beyond the sound line (as shown in Fig. 1(c)) and inside the first Brillouin zone (dashed lines). The dispersion of the surface mode(s) correspond to data that lie outside the sound line, explicitly data with larger magnitude of in-plane momentum, *k*_∥_, than that of a free-space grazing acoustic wave. The dispersion is plotted to values beyond the first Brillouin zone, since the experiment allows for good resolution of the modes far out in momentum-space. All data with momenta values smaller than the sound line correspond to direct sound transmission between the source and microphone detector, or other unwanted noise, and are not discussed.

For the honeycomb array (see Fig. 2(a–c)), experimental results (greyscale data) agree well with numerical simulations (open circles), with one exception; the upper branch of the Dirac cone in the Γ-*K* direction (Fig. 2(a)) is present in the model, but is not observed experimentally in the first Brillouin zone. Conversely, the existence of the upper branch is shown in the *M*-*K* direction for the honeycomb sample (Fig. 2(b)) beyond the *K* point towards Γ at higher momentum, where it meets the lower branch at the Dirac frequency, *f*_D_ = 16.5 kHz. The absence of the upper branch in the Γ-*K* direction within the first Brillouin zone is a consequence of the asymmetry of the mode pressure field with respect to its propagation direction. In an equivalent study of microwave honeycomb structures, Dautova *et al*. show that a non-zero valued reciprocal lattice vector, *G*, is necessary to observe the upper branch of mode above the Dirac frequency^27^, due to a vanishing integral in the Fourier intensity for a mode with an anti-symmetric field distribution. The data in Fig. 2, confirms this analysis; the upper branch is never observed in the first Brillouin zone, but becomes present beyond the Brillouin zone boundary into the second zone (prominently shown in panel (b)).

By plotting the dispersion data (honeycomb) beyond the first Brillouin zone, the modes coming from higher-order diffraction points are clearly seen, being particularly prominent in the *M*-*K* and Γ-*M* directions (Fig. 2(b,c)) as the modes folded back from large positive and negative momentum states cross at the *K* and *M* points. From Fig. 2(a,c) it is evident that the surface mode behaviour is quite different; the mode at *K* clearly has real group velocity (as expected at Dirac-like points), whilst the mode approaching the Brillouin zone at *M* from Γ has zero group velocity producing two standing wave states of different energy. The experimental results for the hexagonal sample (see Fig. 2(d–f)), also show good agreement with the surface mode predicted by numerical simulations, but unlike the honeycomb, the hexagonal sample does not exhibit a Dirac point.

**Figure 3:**
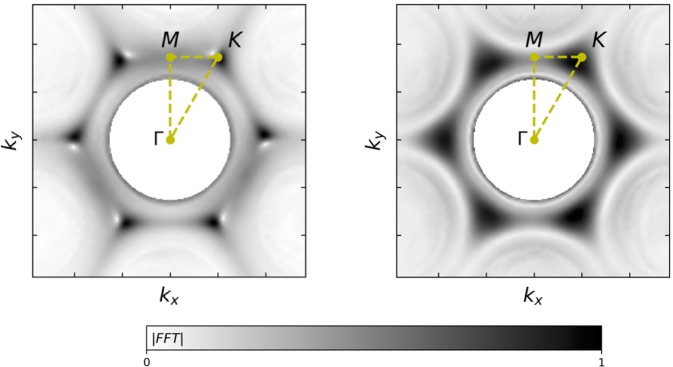
Isofrequency contour plots for (**a**) the honeycomb and the (**b**) hexagonal lattices at the Dirac frequency *f*_D_ = 16.5 kHz. The centre white circle masks all radiated components. The greyscale data shows Fourier intensity normalised to unity.

Further insight into the dispersion of these surface modes is obtained by imaging the dispersion on a momentum plane at a fixed frequency (an isofrequency map), providing information within the planes between points of high symmetry. Figure 3 shows this data at frequency, *f*_D_, corresponding to the Dirac point (in the honeycomb system) for both samples studied. In these graphs, the radiative components are masked by a white circle. At the edge of this circle, there is a dark ring that corresponds to the sound line, beyond which are the surface modes localised at the *K* points.

Due to the real space lattice symmetry of each sample, we might expect the honeycomb isofrequency contour to display six-fold rotational symmetry, however, Fig. 3(a) clearly shows only a three-fold rotational symmetry around the Γ point; this reduction in symmetry arises due to the point source excitation of one cavity of the two-cavity lattice basis. By contrast the hexagonal lattice, Fig. 3(b), does indeed have a six-fold rotational symmetry. For the honeycomb data, at the *K* point there is strong Fourier intensity, with a region of much lower intensity in a direction towards one of the neighbouring diffracted Γ points. Referring back to Fig. 2(b) we see this feature is also present; the surface mode is well coupled from low frequency near *M* and exists through the *K* point and beyond into the second Brillouin zone, and the upper cone is present, but weakly coupled to along the first Brillouin zone between *M* and *K* as dictated by the dashed lines. By looking at the momentum-frequency plane rotated by a multiple of *π*/3 radians around the Γ point, the plane with the other 3-fold symmetry is visualised and the upper edge of the Dirac cone is less well resolved along first Brillouin zone, as shown in Fig. S3.

Experimentally we have shown that both honeycomb and hexagonal arrays of holes in an acoustically rigid material support bound acoustic surface waves. Results show the existence of the Dirac point and the linear dispersion of acoustic surface waves supported by the honeycomb lattice, but not for the similar hexagonal lattice. The modes of the honeycomb lattice at the *K* and *M* high symmetry points are interesting because one exhibits a band gap and the other does not. To further interpret the nature of these modes, computed and measured real-space pressure fields are studied for the honeycomb sample. Such analysis can be used to understand whether a band gap or a crossing of the surface modes exists, and is an approach that has been used to describe dispersion relations in terms of the spatial symmetries of resonance states, for instance, in Sakoda^28^ and in Li *et al*.^29^.

**Figure 4:**
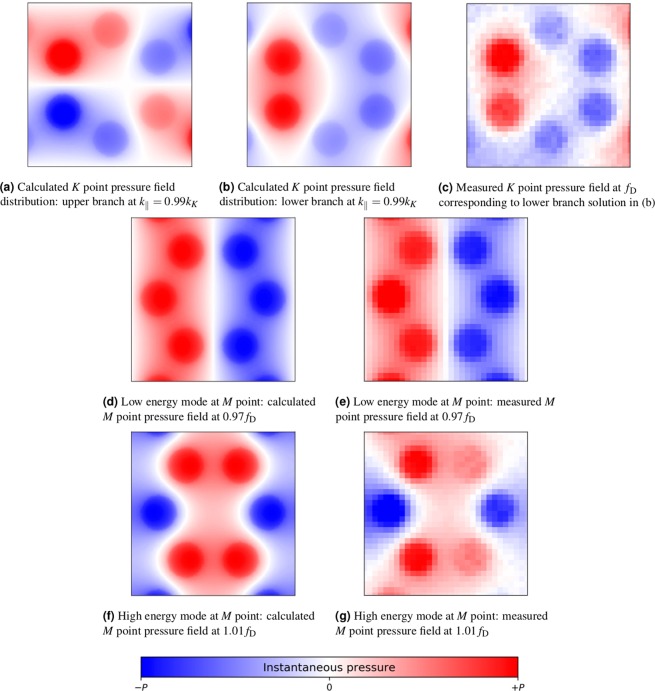
Calculated (frequency- and wavevector-selected) and measured (spatially- and frequency-selected) acoustic pressure fields for the honeycomb structure for modes at *K* and *M* points: (**a–c**) show field distributions at the Dirac frequency for the surface mode propagating along the Γ-*K* direction at the Dirac frequency *f*_D_ (experiment) and *K* point, *k*_*K*_ = 4*π*/3*a* (model). (**d,e**) Show field distributions for the lowest energy mode at frequency *f* = 0.97*f*_D_ (experiment) and *M* point, ${k}_{M}=2\pi /a\sqrt{3}$ (model). (**f,g**) Show equivalent plots the upper mode at *f* = 1.01*f*_D_. Red and blue indicate the maximum positive and negative pressure respectively. Model data is obtained by numerically evaluating the pressure field on the sample surface at the appropriate in-plane wavevector and frequency. The experimental pressure field is by obtained from the Fourier transform of the temporal field measured with the microphone tip approximately 100 *μ*m above the surface over a 15 mm by 15 mm area scan, and selecting the required frequency. The centre coordinates of each measured field map (**c,e,g**) are (*x* [mm], *y* [mm]) = (116.5, −2.5), (5, 62.5) and (0, 55) respectively, relative to the source. Note: figures are orientated so that surface modes would be travelling from left to right on the page.

To image the pressure field distributions for modes near the points of high-symmetry, small area (15 mm by 15 mm) high spatial resolution scans were made. The pressure field distributions are selected by frequency and propagation direction; the experimental data in Fig. 4 is obtained from small area scans and the Fourier analysis of the time domain data only. The pressure field data is then presented at frequencies that correspond to the frequency of the mode at the *K* point and of the upper and lower branch at the *M* point. The small measured areas, measured some distance from the source in either the *x*- or *y*- direction, allow the surface mode field profiles to be selected based on their propagation direction in real space (modes at *K* and *M* are present in real space at 90 degrees to one another). We note that because the modes are selected by frequency and propagation direction - not frequency and wavevector - all wavevectors of that frequency are present in the measured fields, the surface wave being dominant, due to the close proximity of the detector to the surface. The distance from the 2D scan area to the source was a critical factor in recording high quality data that can be usefully interpreted; close to the source the imaged fields are dominated by cylindrical spreading of wavefronts, whilst further away, the surface wave pressure amplitude suffers due to thermo-viscous losses at the sample surface. The *K* point (see Fig. 4) scan was taken approximately 116 mm from the source in the *x*-direction, whilst field maps at the *M* point are taken at approximately half that distance in the orthogonal direction.

Figure 4 compares the calculated and measured fields at *K* and *M* for the honeycomb lattice. Simulated pressure fields are the calculated eigenmodes when the in-plane momentum is equal to the momentum of the symmetry point (with appropriate boundary conditions on the unit cell). Measured pressure fields are presented at the frequency at which that mode meets the Brillouin zone. Figure 4(a,b) show the computed eigenmode solutions close (*k*_||_ = 0.99*k*_*K*_) to the *K* point in order to break the degeneracy of the two solutions when *k*_||_ = *k*_*K*_. The pressure field distribution for the lower branch of mode is symmetric, whereas the pressure field for the upper branch is anti-symmetric with respect to the Γ-*K* vector (the direction of propagation in real space). Commonly in the literature, the fields (pressure or otherwise) of larger scale systems are compared to electron wavefunctions in atomic systems. Here we note these pressure field distributions show great similarity to the so-called *p*_x_ and *p*_y_ ‘photonic orbitals’ of ref.^30^ and are referred to as acoustic pseudo-spin dipolar states in analogous acoustic systems^31^. Apart from visible attenuation of the measure pressure field, from left to right of the figure (Fig. 4(c)), data for the lower branch of the mode agree well with simulations. There is no experimental pressure field configuration for the upper frequency mode since we have not been able to detect this experimentally due to attenuation and the mode group velocity.

The calculated pressure fields at the *M* point band gap in Fig. 4(d,f) for the lower and upper band. These are two standing wave solutions, as expected for a mode at the Brillouin zone with zero group velocity, and have symmetric pressure field distribution with respect to the propagation direction. As before, the experimental pressure field distributions (Fig. 4(e,g)) show good agreement with those predicted by the model. The symmetry of the crystal lattice causes a pair of symmetric Bloch states at the *M* point that result in a band gap, not a Dirac point. Whilst the asymmetry of two degenerate modes at the *K* point are characteristic of the deterministic degeneracy of Bloch states imposed by the symmetry of the crystal lattice, and have been studied in related acoustic systems^12^.

As alluded to above, in the experimental field maps and in Fig. S2, the attenuation of these acoustic surface modes is significant at the frequencies studied here. For instance, the pressure field distribution in Fig. S2(c) clearly shows that the surface mode is highly localised to the source, as the mode approaches the Dirac frequency where the mode group velocity approaches zero and loss is significant. Recent studies have explored such losses by implementing Laplace transform techniques in order to retrieve the imaginary component of the wavevector^32^. Here, the attenuation of the modes approaching or travelling through the *K* point is quantified through the width of the mode in momentum. Data is fit at each frequency by a skewed Lorentzian distribution for modes propagating with positive and negative momentum and the full-width at half-maximum (FWHM) is estimated (see *Methods* for further details).

**Figure 5:**
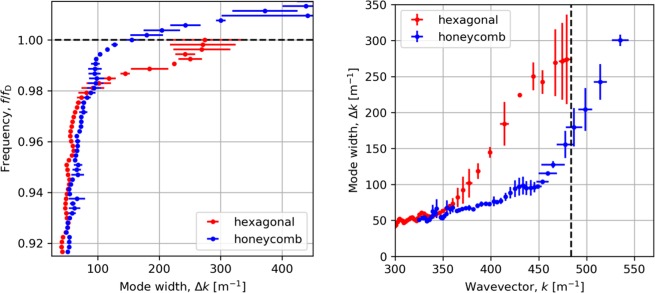
Analysis of acoustic surface wave loss as a function of frequency (left) and wavevector (right) for mode propagating in the Γ to *K* plane for honeycomb and hexagonal lattices. Data points are the mean values from the fit of forward and backward travel modes; error bars span the difference in fit values.

The losses (widths) are shown in Fig. 5 for modes propagating on both the honeycomb and hexagonal lattice. We notice that the mode width increases with frequency monotonically for both lattices, which is consistent with the mode group velocity (▽_*k*_*ω*). The mode propagating on the honeycomb lattice moves through the Dirac point without any perturbation to its width. In contrast, the mode on the hexagonal lattice is strongly perturbed by the lattice and becomes a standing wave at the *K* point. As the mode meets the Brillouin zone the mode width, Δ*k* and momentum, *k*, tend to a fixed value. This confirms the expected nature of the modes; namely one propagating and one stopping through/at the *K* symmetry point.

## Conclusions

In summary, we have studied the dispersion relations and the pressure field configurations of acoustic surface waves on a honeycomb array and on a hexagonal array of open holes using both experiment and numerical simulations. For the honeycomb array, experimental results show that (for the lowest order cavity resonance) two acoustic surface modes exist, where at the *K* point they exhibit a Dirac point, and at the *M* point a band gap is seen. Numerical simulations were performed that show close agreement with the data obtained experimentally; confirming the Dirac point at *K* and a band gap at the *M* point. Moreover, it is shown that for the honeycomb sample the losses increase with increasing momentum along the Γ-*K* direction, reflecting the propagation of acoustic surface waves with diminishing group velocity. The measured dispersion relation in the Γ-*K* direction and in the isofrequency data, shows the excitation and detection of surface modes is dependent on the source conditions and that the mode symmetry can determine whether it is observable in the first Brillouin zone; in our measurement this manifested itself in the absence of the upper branch in the Γ-*K* direction in the first Brillouin zone, and may be the reason for partial resolution of the same mode in the previously reported measurements of acoustic graphene^23^.

The results for the honeycomb lattice have also been compared with those from a hexagonal array. The hexagonal array was chosen to have the same unit cell dimensions in real space, to provide a comparator in momentum space. Results showed that only one lower order surface mode exists, that it occurs across similar frequencies to its honeycomb counterpart, but does not show Dirac-like behaviour. Analysis of the acoustic pressure fields at the *K* and *M* points of high lattice symmetry for the honeycomb array demonstrate the field symmetries for the Dirac point and the band gap.

Using a simple fitting-approach to estimate the mode loss we see that the mode that travels through the Dirac point from the first into the second Brillouin zone is unperturbed around the *K*-point. These results highlight the importance of excitation conditions and the acoustic loss of surface modes on structured surfaces on the realisation of designs that exploit surface modes at audible frequencies in the kilohertz range.

## Methods

### Numerical modelling

Surface mode dispersion was calculated using the Finite Element Method (FEM) modelling package, COMSOL Multiphysics (version 5.3a)^33^. The dispersion relations presented are the eigenmodes of rhombic unit cells with repeat Floquet-periodic boundary used to represent an infinite surface. These unit cells are displayed in Fig. 1(a,b). The model assumes the holes are cylindrical cavities perforated through an acoustically-rigid plate of thickness, *t*. The acoustic loss that arises due to the no-slip and isothermal boundary condition at the fluid-solid interfaces are accounted for (implemented using the pressure acoustic and thermo-acoustic modules); for the geometries and frequencies studied here, loss causes a small (approx. 0.5 kHz) reduction in the frequency of the modes at the Brillouin zone when compared a loss-less system.

### Sample manufacture

Experimental samples (photograph in Fig. 1) were fabricated in aluminium alloy by milling each hole array geometry through plates of mean thickness *t* = 8.0 ± 0.1 mm. The hole array structures occupy a 400 mm by 490 mm area on the plate surface.

### Acoustic measurements

Acoustic measurements of the surface wave pressure field used a fixed loudspeaker source and a detector on the opposite side of the sample on a translation stage. Figure S1 displays a simple schematic of this experimental setup. Acoustic surface modes were excited using a modified Tucker-Davis Technologies MF1 near-field source positioned over a hole in the centre of the sample to diffractively couple sound to the surface modes. This source was excited with single cycle sine-Gaussian waveform with 16 kHz carrier frequency. Surface wave propagation across the sample is measured with a Brüel & Kjær Type 4182 needle-tip probe microphone positioned on the opposite side of the sample and raster scanned in a plane approximately <0.4 mm above sample surface using the translation stage. At each spatial point the time-dependent signal (voltage) is recorded with a sampling frequency of 312.5 kHz, for a duration of 32 ms. The detecting microphone was scanned in square grid with raster step size, Δ*x* = Δ*y*, of 1.5 mm, for a total scan length, *x*_max_ = *y*_max_, of 400 mm. The momentum-space resolution of this experiment is limited by the scan length, Δ*k* = 1/*x*_max_, and the range is limited by minimum scan step size, Δ*x* (maximum wavevector *k*_max_ = 2*π*/Δ*x*). The frequency resolution is determined in the same way.

### Experimental data analysis

Fourier analysis of the acoustic signals in time (*t*) and space (*x*, *y*) was used to obtain the full dispersion relation (*k*_x_, *k*_y_, *f*) and pressure field of the surface waves measured close to the sample surface. The dispersion plots shown in Figs 2 and 3 are found by intersecting the full dispersion relation (*k*_x_, *k*_y_, *f*) with an appropriate 2D plane; for plots in Fig. 2 (*k*_||_ vs. *f*) intersections are made to map the Γ-*X*, Γ-*M*, and *M*-*K* directions as a function of frequency, and Fig. 3 (*k*_x_ vs. *k*_y_) to show intersections of the *k*_x_-*k*_y_ plane at a fixed frequency.

All dispersion data presented has undergone real-space windowing of the complex pressure field using a symmetric tapered cosine-window with shape parameter, *α* = 0.5 before being Fourier transformed and zero-padded by a factor of 3. Dispersion curves are plotted with in greyscale indicating the Fourier intensity without normalisation to show the relative strength of features within the plots. All data was processed using the SciPy and NumPy packages and plotted using Matplotlib in Python. To provide an indication of the surface mode loss, the momentum, *k*, and momentum width, Δ*k* of the modes was found by fitting the asymmetric Lorentzian peaks^34^. Peaks were fit (using the curve_fit function in the Python SciPy optimise library) to the mode propagating in the positive and negative *k*-directions independently and results averaged.

## Supplementary information

Supplementary Information
