# Supplementary material for: Experimental characterisation of the bound acoustic surface modes supported by honeycomb and hexagonal hole arrays
Source: Sci Rep. 2019 Oct 31;9:15773. doi: 10.1038/s41598-019-50446-z (PMC6823496; doi:10.1038/s41598-019-50446-z)
Supplement: Supplementary file 1 — Supplementary Information [file 41598_2019_50446_MOESM1_ESM.pdf]

Experimental characterisation of the bound acoustic surface modes supported by honeycomb and hexagonal hole arrays

Timothy A. Starkey[1,*], **Vicky Kyrimi**[1], **Gareth P. Ward**[1], **J. Roy Sambles**[1], **and Alastair P. Hibbins**[1]

[1]University of Exeter, Electromagnetic and Acoustic Materials Group, Department of Physics and Astronomy, Exeter, EX4 4QL, United Kingdom
[*]t.a.starkey@exeter.ac.uk

Supplementary material

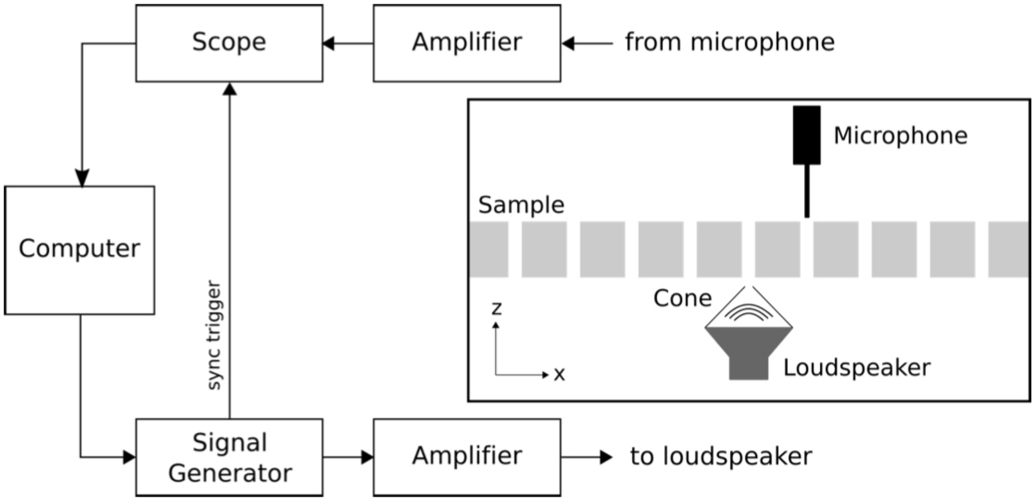

Figure S1. A simple schematic diagram of the experimental method.

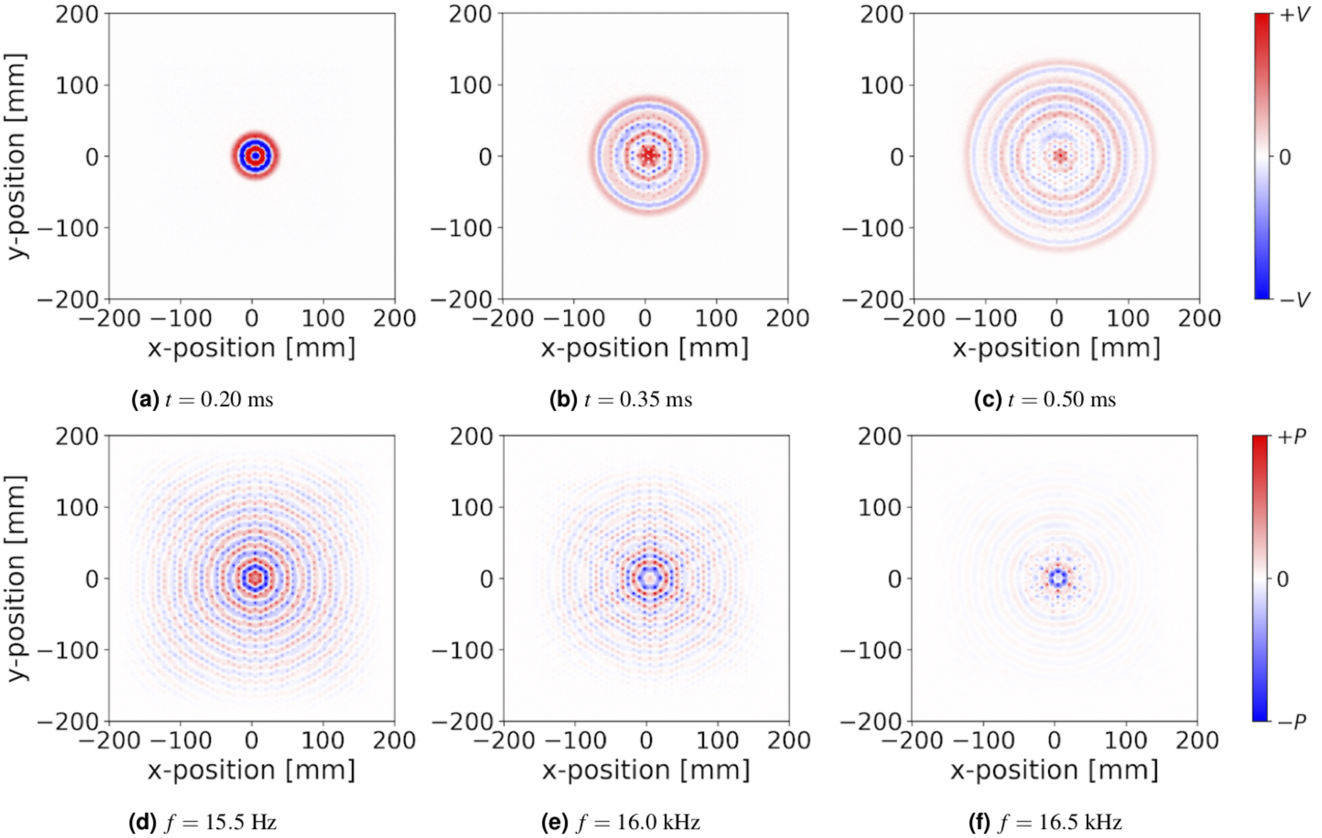

(a) $t = 0.20$ ms **(b)** $t = 0.35$ ms **(c)** $t = 0.50$ ms

(d) $f = 15.5$ Hz **(e)** $f = 16.0$ kHz **(f)** $f = 16.5$ kHz

Figure S2. Exemplar experimental data for the hexagonal lattice measurement: panels (a) - (c) display the signal (voltage) map of the acoustic pulse propagating across the hexagonal lattice at three times, t, after the pulse is triggered from a source at the centre of the sample. Panels (d) - (f) display the instantaneous pressure field for three different frequencies, f, which visually shows the 6-fold lattice symmetry.

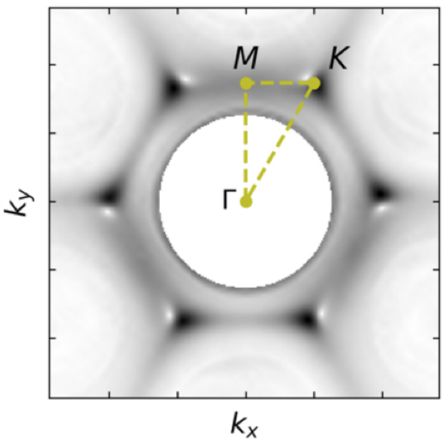

(a) Isofrequency map at the Dirac frequency, f_D = 16.5 [kHz]

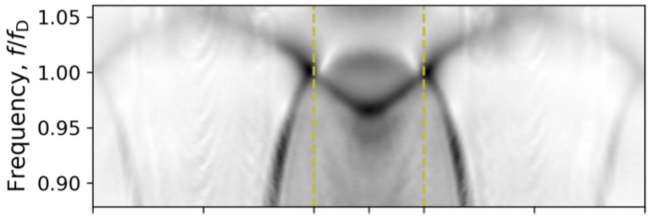

(b) *M*-*K* dispersion diagram for positive k_y

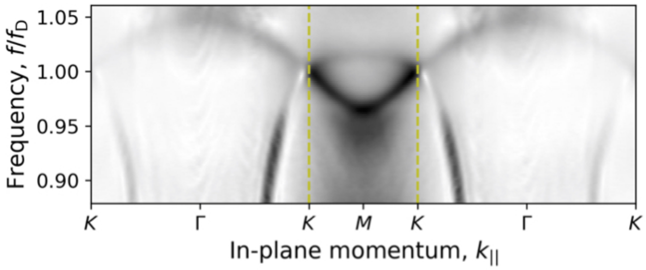

(c) *M*-*K* dispersion diagram for negative k_y

Figure S3. Dispersion data for honeycomb lattice demonstrating the 3-fold symmetry related to the coupling of the point source: (a) shows isofrequency map at the Dirac frequency, (b) and (c) displays dispersion diagrams for the *M*-*K* lattice plane at the first Brillouin zone for positive and negative k_y respectively (as dictated by panel (a)).
